# Efficacy and Safety of Avatrombopag in Patients with Chronic Liver Disease and Thrombocytopenia Undergoing Elective Surgery

**DOI:** 10.3390/jcm15145715

**Published:** 2026-07-21

**Authors:** Weihua Cao, Fengxin Chen, Hongxiao Hao, Xin Wei, Xinxin Li, Ziyu Zhang, Wen Deng, Shiyu Wang, Linmei Yao, Zixuan Gao, Shuojie Wang, Lu Zhang, Yao Lu, Yuanjiao Gao, Yao Xie, Minghui Li

**Affiliations:** 1 Department of Hepatology Division 2, Beijing Ditan Hospital, Capital Medical University, Beijing 100015, China; weihuacaohappy@163.com (W.C.);; 2 HBV Infection, Clinical Cure and Immunology Joint Laboratory for Clinical Medicine, Capital Medical University, Beijing 100015, China; 3 Department of Hepatology Division 2, Peking University Ditan Teaching Hospital, Beijing 100015, China

**Keywords:** avatrombopag, chronic liver disease, thrombocytopenia, efficacy, safety

## Abstract

**Background/Objectives**: The aim of this study was to evaluate the efficacy and safety of avatrombopag in patients with chronic liver disease (CLD) and thrombocytopenia scheduled for elective invasive/minimally invasive procedures, providing clinical guidance for patients requiring platelet (PLT) elevation. **Methods**: In this single-center, prospective study, patients with CLD and PLT counts <50 × 10^9^/L were scheduled for elective invasive/minimally invasive surgery, receiving 5-day avatrombopag plus standard CLD management. PLT response, dynamics, and safety were assessed. **Results**: A total of 108 patients with CLD and baseline PLT counts <50 × 10^9^/L were enrolled, showing an 83.33% response rate. Responders exhibited significantly higher baseline white blood cell (WBC, *p* = 0.009), neutrophil (*p* = 0.016), hemoglobin (HGB, *p* = 0.011), and PLT (*p* = 0.001) levels compared to non-responders. Baseline PLT correlated positively with age (*p* = 0.014), WBC (*p* = 0.046), HGB (*p* = 0.001), and prothrombin activity (*p* = 0.023). Logistic regression identified baseline PLT as an independent predictor of treatment response (*p* = 0.002). PLT began rising by day 5 post-treatment, peaked around day 10, and declined to baseline by day 40 in overall cases and responders. Non-responders showed only mild PLT elevation by day 5 (remaining <50 × 10^9^/L), with no further increase by day 10. No adverse events were observed. No thrombotic or bleeding events were recorded in this small cohort; however, the limited sample size precludes definitive conclusions on thrombosis risk. **Conclusions**: Avatrombopag demonstrated high efficacy and favorable safety for elevating PLT in patients with CLD, with a higher baseline PLT predicting a better response.

## 1. Introduction

Thrombocytopenia frequently complicates chronic liver disease (CLD), arising from multifactorial mechanisms including reduced hepatic thrombopoietin (TPO) synthesis, hypersplenism secondary to portal hypertension, bone marrow suppression, and immune-mediated platelet destruction. The reported prevalence ranges from 6% to 78% depending on the severity of liver disease, and severe thrombocytopenia (platelet (PLT) counts <50 × 10^9^/L) is associated with an increased bleeding risk during invasive procedures [1,2,3]. Giannini et al. [4] reported that the prevalence of thrombocytopenia exceeded 76% among patients with cirrhosis. Although spontaneous bleeding is uncommon in patients with CLD and thrombocytopenia, PLT counts <50 × 10^9^/L significantly increase perioperative bleeding risks during invasive or minimally invasive procedures such as liver biopsy, endoscopic variceal ligation, or tumor ablation, and may even preclude surgical feasibility [1,3,5]. Prophylactic PLT transfusion remains the primary intervention for severe thrombocytopenia in elective invasive procedures. Although it temporarily elevates PLT, its limitations—including donor dependency, infection risks, immune reactions, and short-lived efficacy—restrict clinical utility [3,6,7,8], highlighting the need for safer alternatives.

TPO, a multifunctional cytokine predominantly produced by the liver, bone marrow, and kidneys, regulates megakaryocyte and platelet production. By binding to TPO receptors (TPO-R) on stem cells, megakaryocyte progenitors, mature megakaryocytes, and platelets, TPO modulates megakaryocyte proliferation, maturation, and PLT release throughout developmental stages. As the most critical growth factor for megakaryocyte development and thrombopoiesis, TPO synthesis is primarily hepatic and renal [9].

Avatrombopag, a second-generation oral non-peptide thrombopoietin receptor agonist (TPO-RA), stimulates platelet production via TPO-R activation on bone marrow megakaryocytes. Its advantages include no dietary restrictions, convenient weekly dosing, and minimal hepatotoxicity [10,11]. Two pivotal phase III trials (ADAPT-1 and ADAPT-2) demonstrated that avatrombopag significantly elevates PLT in patients with CLD, enabling 84–88% to achieve PLT counts ≥50 × 10^9^/L on procedure day without platelet transfusions, outperforming a placebo (66% vs. 23%) [3,10]. It exhibits favorable safety, with headache, fatigue, and gastrointestinal adverse events comparable to a placebo and no increased thrombosis risk [10,12,13]. However, the existing studies predominantly involve Western populations, with limited efficacy and safety data for East Asian patients, particularly those with hepatocellular carcinoma or portal vein tumor thrombosis [1,10]. Its real-world applicability for complex surgeries (e.g., hepatectomy, liver transplantation), synergy with anticancer therapies, and long-term risks (e.g., thrombosis) requires further validation [10,14,15]. Additionally, optimal dosing strategies for minimally invasive procedures (e.g., percutaneous radiofrequency ablation) and comparative efficacy with similar agents (e.g., lusutrombopag) lack high-quality evidence [5,7,16,17]. Genetic variants may be associated with CLD, such as NAFLD [18]. One study used an artificial neural network (ANN) model combined with dietary retinol intake from different sources to predict the risk of CLD [19].

This prospective observational study aimed to evaluate PLT response dynamics, PLT trends, and safety outcomes in Chinese patients with CLD and severe thrombocytopenia receiving avatrombopag before elective invasive/minimally invasive procedures. The findings will provide clinical guidance for PLT elevation and generate high-quality real-world evidence to optimize perioperative management.

## 2. Materials and Methods

### 2.1. Study Design

This was a single-center, prospective, observational real-world study conducted at the Department of Hepatology Division 2, Beijing Ditan Hospital, Capital Medical University, China. Avatrombopag was prescribed as part of routine clinical practice based on the treating physician’s independent clinical judgment, in accordance with its approved indication in China for thrombocytopenia in patients with chronic liver disease scheduled for elective invasive procedures. This study did not mandate or assign the use of avatrombopag; rather, it prospectively observed and recorded clinical outcomes in patients who received avatrombopag as part of their standard perioperative management. Eligibility for treatment, dosage selection (60 mg/day for PLT counts < 40 × 10^9^/L, 40 mg/day for PLT 40–50 × 10^9^/L), the standard treatment regimen of 5 consecutive days of oral administration, and the timing of procedures (10 ± 3 days after treatment initiation) reflected routine clinical practice and were aligned with the drug’s pharmacokinetic profile and prescribing information. Follow-up visits and data collection time points (baseline, day 5, day 10, and day 40) were standardized for the purposes of systematic data capture in this observational study. This study is reported in accordance with the STROBE guidelines for observational studies and is classified as an observational (non-interventional) study.

### 2.2. Study Population

Chronic liver disease was defined based on clinical, biochemical, imaging, and/or histopathological evidence of liver cirrhosis or chronic hepatitis for at least 6 months. Patients with CLD and thrombocytopenia scheduled for elective invasive or minimally invasive procedures at the Department of Hepatology Division 2 of Beijing Ditan Hospital, Capital Medical University, from June 2024 to June 2025 were enrolled.

Inclusion Criteria: (1) Voluntary participation with signed informed consent. (2) Age ≥ 18 years, regardless of gender. (3) Baseline PLT count <50 × 10^9^/L. (4) Patients with chronic liver disease scheduled for elective invasive/minimally invasive procedures. (5) No fertility requirements.

Exclusion Criteria: (1) History of arterial/venous thrombosis within 6 months prior to baseline, (2) Platelet transfusion or platelet-containing blood products within 7 days before baseline. (3) Inability to discontinue anticoagulant/antiplatelet therapy preoperatively (low-dose aspirin permitted). (4) Current treatment with recombinant thrombopoietin (rhTPO) or thrombopoietin receptor agonists (e.g., eltrombopag, romiplostim). (5) Severe atherosclerosis, predisposition to cerebral thrombosis, or coronary stenosis > 70%. (6) History of arterial/venous thrombosis within 6 months prior to enrollment. (7) Portal vein flow velocity < 10 cm/s or prior portal vein thrombosis within 6 months. (8) Primary hematologic disorders (e.g., immune thrombocytopenia, myelodysplastic syndrome, aplastic anemia). (9) Inherited thrombophilia (e.g., Factor V Leiden mutation, prothrombin G20210A mutation, hereditary ATIII deficiency). (10) Major cardiovascular events within 6 months (e.g., worsening heart failure, arrhythmias increasing thromboembolic risk [e.g., atrial fibrillation], coronary/peripheral stenting/angioplasty, or bypass surgery). (11) Hypersensitivity to avatrombopag or its excipients. (12) Psychiatric disorders or vulnerable populations with impaired decision-making capacity. (13) Comorbidities deemed by investigators to jeopardize patient safety.

This study was approved by the Ethics Committee of Beijing Ditan Hospital, Capital Medical University (NO. DTEC-KY2024-043-02, and Approval Date: 15 June 2024) and was registered in the Clinical Trials (NCT06642740). All patients provided written informed consent.

### 2.3. Treatment Protocol

Avatrombopag was administered in addition to standard liver disease therapy. Patients with PLT counts <40 × 10^9^/L received 60 mg/day, while those with counts 40–50 × 10^9^/L received 40 mg/day, both for 5 consecutive days. Invasive/minimally invasive procedures were scheduled for 10 (±3) days after treatment initiation.

### 2.4. Patient Follow-Up

Platelet counts were measured at the following time points: (i) baseline (day 0, immediately before avatrombopag initiation); (ii) treatment day 5 (±1 day), i.e., the day after the last dose of avatrombopag; (iii) procedure day, which was planned to be 10 (±3) days after treatment initiation (i.e., day 10 on average); and (iv) follow-up day 40 (±3 days) after procedure. All blood samples were drawn between 7:00–9:00 AM after an overnight fast.

Monitoring Parameters: (a) Clinical biochemistry (ALT, AST, total bilirubin, direct bilirubin, GGT), complete blood count, coagulation profile (prothrombin activity, INR), blood glucose, and lipids. (b) Adverse events during follow-up.

### 2.5. Evaluation Metrics

Primary Endpoint: A proportion of patients achieving PLT counts ≥50 × 10^9^/L on the procedure day.

Secondary Endpoints: Incidence of adverse events, particularly short- and long-term thrombotic/bleeding events.

### 2.6. Statistical Analysis

Continuous variables were described using counts, means, standard deviations, medians, minima, and maxima. Categorical variables were summarized as frequencies and percentages. Intergroup comparisons used *t*-tests, Mann–Whitney U tests, chi-square tests, or Fisher’s exact tests. Ordinal data were analyzed via Wilcoxon rank-sum tests. Safety assessments included adverse event frequency, duration, severity, causality, and outcomes, alongside a descriptive analysis of laboratory changes. For the correlation analysis, we used Spearman’s rank correlation for non-normally distributed variables (as most continuous data were not normal) and Pearson correlation for normally distributed variables (after Shapiro–Wilk test). This is now clearly stated. For logistic regression, we first performed univariate analysis for all the baseline variables listed in Table 1. Those with *p* < 0.10 in the univariate analysis (WBC, HGB, PLT, and also NE with *p* = 0.093) were entered into a stepwise forward selection multivariate model, with the entry criterion *p* < 0.05 and removal *p* > 0.05. The final model retained only PLT as a significant independent predictor. Analyses were performed using IBM SPSS Statistics 26.0, with two-tailed tests (α = 0.05) and 95% confidence intervals.

## 3. Results

### 3.1. Baseline Characteristics of Avatrombopag Responders vs. Non-Responders in Patients with CLD and PLT Counts <50 × 10^9^/L Undergoing Elective Invasive/Minimally Invasive Surgery

**Figure 1:**
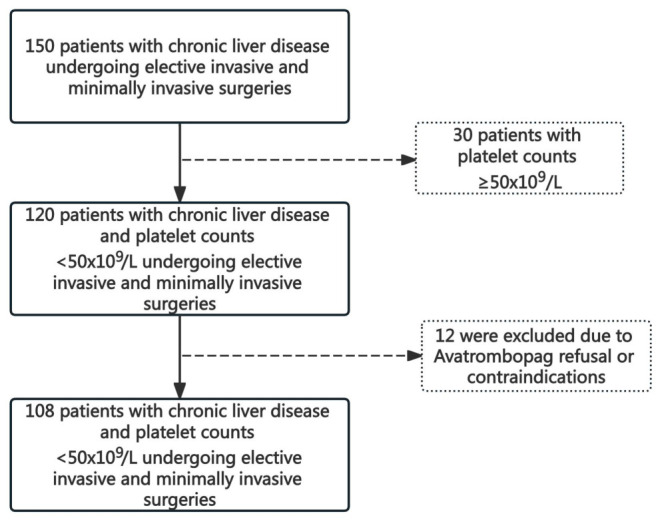
Patients’ enrollment flowchart.

All patients were enrolled from the Hepatology Department of Beijing Ditan Hospital and required elective invasive/minimally invasive procedures with baseline PLT counts <50 × 10^9^/L. The etiology included hepatitis B virus (HBV) infection (*n* = 72, 66.7%), hepatitis C virus (HCV) (*n* = 12, 11.1%), alcohol-related liver disease (*n* = 14, 13.0%), non-alcoholic fatty liver disease (NAFLD, *n* = 6, 5.6%), and others (*n* = 4, 3.7%). Cirrhosis was confirmed by transient elastography (liver stiffness ≥ 12.5 kPa) or abdominal ultrasound/CT/MRI showing a nodular liver surface, splenomegaly, and/or signs of portal hypertension. The Child–Pugh class distribution was: A in 56 patients (51.9%), B in 43 (39.8%), and C in 9 (8.3%). Patients who met the inclusion criteria and received avatrombopag (Figure 1) were included for clinical observation. All patients continued standard liver disease therapy and received avatrombopag for 5 days (conventional treatment). Baseline demographic data, biochemical parameters, and post-treatment biochemical indices were collected. The proportion of patients achieving PLT counts ≥50 × 10^9^/L on the procedure day and the incidence of adverse events were analyzed. In our study, the elective invasive/minimally invasive procedures included: liver biopsy (*n* = 32), endoscopic variceal ligation/endoscopic therapy (*n* = 28), percutaneous radiofrequency ablation or microwave ablation for hepatocellular carcinoma (*n* = 26), transcatheter arterial chemoembolization (*n* = 15), and paracentesis or other abdominal interventions (*n* = 7) (the total exceeds 108 because some patients underwent multiple procedures simultaneously).

As is shown in Table 1, 108 patients with CLD (mean age: 55.69 ± 1.26 years; 74 males [68.52%], 34 females [31.48%]) were included. Among them, 90 patients (83.33%) responded to avatrombopag, while 18 (16.67%) were non-responders. No significant differences in gender or age were observed between the responders and non-responders. The responders had significantly higher baseline white blood cell (WBC, *p* = 0.009), neutrophil (NE, *p* = 0.016), hemoglobin (HGB, *p* = 0.011), and PLT (*p* = 0.001) levels compared to the non-responders. However, no significant differences were found in baseline ALT, AST, TBIL, DBIL, ALB, GGT, triglyceride, HDL, LDL, blood urea nitrogen, serum creatinine, serum uric acid, blood glucose, PTA, or INR levels between groups (Table 1).

**Table 1:** Comparison of clinical characteristics between responder group and non-responder group.

Item	All Patients *n* = 108	Responder Group *n* = 90	Non-Responder Group *n* = 18	t or Z, χ^2^	*p* Value
Sex Male (%)	74 (68.52%)	64 (71.11%)	10 (55.56%)	1.683	0.195
Age, mean ± SD (years)	55.69 ± 1.26	55.29 ± 11.01	57.72 ± 8.35	0.887	0.377
WBC, median [Q1, Q3] (×10^9^/L)	2.55 (1.67, 3.11)	2.61 (1.87, 3.12)	1.70 (1.23, 2.58)	2.605	** 0.009
NE#, median [Q1, Q3], (×10^9^/L)	1.38 (0.94, 2.00)	1.43 (1.09, 2.05)	1.02 (0.80, 1.31)	2.415	* 0.016
HGB, mean ± SD, (g/L)	100.89 ± 28.90	104.03 ± 29.01	85.17 ± 23.17	2.595	* 0.011
PLT, median [Q1, Q3], (×10^9^/L)	39.50 (30.25, 44.00)	40.00 (33.00, 45.00)	24.00 (15.25, 40.00)	3.181	*** 0.001
ALT, median [Q1, Q3] (U/L)	21.25 (16.90, 30.33)	22.05 (17.73, 30.78)	17.90 (14.18, 27.15)	1.830	0.067
AST, median [Q1, Q3] (U/L)	27.85 (21.65, 35.43)	28.70 (22.03, 36.75)	23.70 (21.25, 29.85)	1.690	0.091
TBIL, median [Q1, Q3] (umol/L)	22.00 (15.30, 33.18)	22.45 (14.45, 33.48)	19.95 (17.70, 33.08)	0.148	0.882
DBIL, median [Q1, Q3] (umol/L)	9.45 (6.53, 13.58)	9.30 (6.33, 13.60)	9.85 (8.10, 12.83)	0.660	0.510
ALB, median [Q1, Q3] (g/L)	33.55 (30.70, 37.10)	33.95 (30.78, 37.30)	32.45 (30.23, 34.58)	1.356	0.175
GGT, median [Q1, Q3] (U/L)	31.45 (17.45, 66.88)	33.15 (20.08, 68.18)	19.35 (12.10, 61.73)	1.896	0.058
Triglycerides, median [Q1, Q3] (mmol/L)	0.77 (0.47, 1.12)	0.78 (0.53, 1.14)	0.60 (0.42, 1.12)	0.989	0.322
HDL, mean ± SD (mmol/L)	1.10 ± 0.34	1.09 ± 0.34	1.16 ± 0.35	−0.865	0.389
LDL, median [Q1, Q3] (mmol/L)	1.57 (1.24, 2.11)	1.57 (1.23, 2.13)	1.57 (1.24, 1.93)	0.548	0.584
Blood urea nitrogen, median [Q1, Q3] (mmol/L)	5.71 (5.08, 6.63)	5.69 (5.06, 6.65)	6.07 (5.12, 6.71)	−0.482	0.630
Serum creatinine, median [Q1, Q3] (umol/L)	65.30 (59.23, 76.53)	65.65 (59.68, 75.58)	64.20 (55.95, 79.50)	0.301	0.763
Serum Uric acid, median [Q1, Q3] (umol/L)	324.50 (242.25, 388.00)	322.00 (240.15, 384.00)	340 (278.75, 393.75)	−0.767	0.443
Blood glucose, mean ± SD (mmol/L)	5.26 (4.81, 6.56)	5.28 (4.80, 6.71)	5.23 (4.83, 6.19)	0.420	0.674
PTA, mean ± SD (%)	65.40 ± 15.98	65.57 ± 16.44	64.56 ± 13.84	0.244	0.808
INR, median [Q1, Q3]	1.34 (1.22, 1.50)	1.32 (1.22, 1.51)	1.39 (1.31, 1.50)	1.043	0.297

Note: WBCs, white blood cells; NEs, neutrophils; HGB, hemoglobin; PLT, platelet; ALT, alanine aminotransferase; AST, aspartate aminotransferase; TBIL, total bilirubin; DBIL, direct bilirubin; GGT, glutamyl transferase; HDL, high-density lipoprotein; LDL, low-density lipoprotein; PTA, prothrombin activity; INR, international normalized ratio. * *p* < 0.05, ** *p* < 0.01, *** *p* < 0.001.

### 3.2. Correlation Analysis Between Baseline PLT Levels and Clinical Parameters

**Figure 2:**
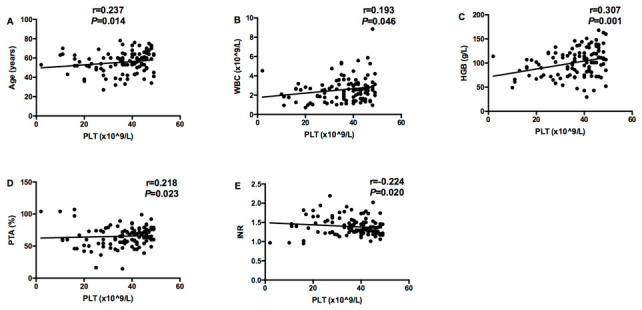
Correlation between platelet levels and age, blood routine, and coagulation function parameters. (**A**). Correlation between platelet levels and age. (**B**). Correlation between platelet levels and WBC. (**C**). Correlation between platelet levels and HGB level. (**D**). Correlation between platelet levels and PTA. (**E**). Correlation between platelet levels and INR.

**Figure 3:**
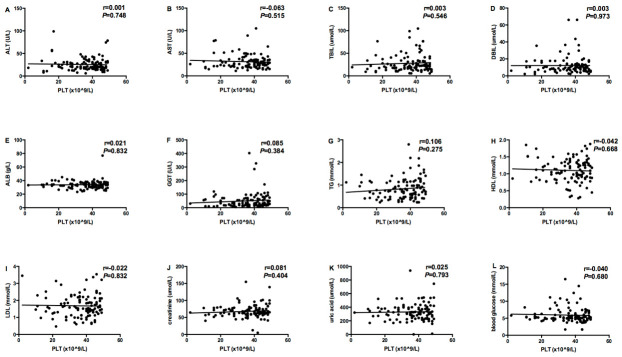
No correlation between platelet levels and liver and kidney function indicators. (**A**). Correlation between PLT levels and ALT levels. (**B**). Correlation between PLT levels and AST levels. (**C**). Correlation between PLT levels and TBIL levels. (**D**). Correlation between PLT levels and DBIL levels. (**E**). Correlation between PLT levels and ALB levels. (**F**). Correlation between PLT levels and GGT levels. (**G**). Correlation between PLT levels and TG levels. (**H**). Correlation between PLT levels and HDL levels. (**I**). Correlation between PLT levels and LDL levels. (**J**). Correlation between PLT levels and creatinine levels. (**K**). Correlation between PLT levels and uric acid levels. (**L**). Correlation between PLT levels and blood glucose levels.

Baseline PLT levels showed positive correlations with age (*p* = 0.014), WBC (*p* = 0.046), HGB (*p* = 0.001), and prothrombin activity (PTA, *p* = 0.023), and a negative correlation with the international normalized ratio (INR, *p* = 0.020) in Figure 2. However, there were no correlations between PLT levels and ALT, AST, TBIL, DBIL, ALB, GGT, triglyceride, HDL, LDL, blood urea nitrogen, serum creatinine, serum uric acid, and blood glucose levels, as shown in Figure 3.

### 3.3. Independent Factors Influencing Avatrombopag Efficacy

Univariate logistic regression identified WBC (*p* = 0.023), HGB (*p* = 0.014), and PLT (*p* < 0.001) as predictors of treatment response. Multivariate analysis confirmed baseline PLT as the sole independent predictor of efficacy (*p* = 0.002) in Table 2.

We have now performed a ROC curve analysis to determine the optimal baseline PLT cut-off for predicting response. The area under the curve (AUC) was 0.738 (95% CI: 0.597–0.879). The Youden index indicated an optimal cut-off of 32.5 × 10^9^/L, with a sensitivity of 74.4% and specificity of 72.2% for predicting responses. This cut-off value supports our clinical suggestion that patients with a baseline PLT count ≥30 × 10^9^/L are more likely to benefit.

### 3.4. Dynamic Changes in PLT Counts Before and After Avatrombopag Treatment

**Figure 4:**
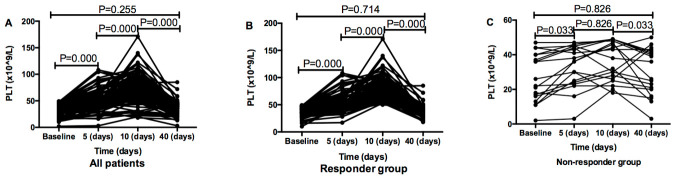
Changes of platelet counts in patients pre- and post-treatment with avatrombopag. (**A**). Changes of platelet counts in all patients pre- and post-treatment with avatrombopag. (**B**). Changes of platelet counts in response patients pre- and post-treatment with Avatrombopag. (**C**). Changes of platelet counts in non-response patients pre- and post-treatment with Avatrombopag.

PLT levels were analyzed in 108 patients before and after treatment with avatrombopag. On day 5 of treatment, the PLT levels were significantly higher than the baseline levels (48.00 vs. 39.50, *p* = 0.000). By follow-up day 10, the PLT levels had increased significantly compared to day 5 (69.50 vs. 48.00, *p* = 0.000). At follow-up day 40, the PLT levels were significantly lower than those on follow-up day 10 (41.00 vs. 69.50, *p* = 0.000), but showed no significant difference compared to the baseline levels (41.00 vs. 39.50, *p* = 0.255), as illustrated in Figure 4A. The trend of PLT level changes in the overall population of 108 patients before and after avatrombopag treatment demonstrated that PLT levels began to rise by day 5 of treatment, peaked around follow-up day 10, and subsequently declined. By follow-up day 40, the PLT levels had returned to pre-treatment levels, as shown in Figure 4A.

We further conducted statistical analysis on the changes of PLT levels before and after therapy in the avatrombopag effective and ineffective groups. The results of the effective group showed that the PLT levels on day 5 of treatment were significantly higher than the baseline levels (50.00 vs. 40.00, *p* = 0.000), as shown in Figure 4B. The PLT levels at follow-up day 10 were significantly higher than those on treatment day 5 (76.00 vs. 50.00, *p* = 0.000), while the PLT levels at follow-up day 40 were significantly lower than those at follow-up day 10 (41.00 vs. 76.00, *p* = 0.000), as seen in Figure 4B. No significant difference was observed between follow-up day 40 PLT levels and the baseline levels (41.00 vs. 40.00, *p* = 0.714), as shown in Figure 4B. Analysis of the 90 avatrombopag-responsive patients revealed the following trend: PLT levels began to rise by treatment day 5, peaked around follow-up day 10, then gradually declined, returning to baseline levels by follow-up day 40, as shown in Figure 4B.

In the ineffective group, PLT levels showed a mild increase on treatment day 5 compared to baseline (36.00 vs. 24.00, *p* = 0.033), as shown in Figure 4C. No significant difference was observed between follow-up day 10 and treatment day 5 levels (35.00 vs. 36.00, *p* = 0.826). The PLT levels at follow-up day 40 were lower than those at follow-up day 10 (31.00 vs. 35.00, *p* = 0.033), with no significant difference compared to the baseline levels (31.00 vs. 24.00, *p* = 0.826), as illustrated in Figure 4C. Analysis of the 18 non-responding patients demonstrated that PLT levels exhibited a mild elevation by treatment day 5 (remaining below 50 × 10^9^/L), failed to show further increase by follow-up day 10, and subsequently declined to baseline levels by follow-up day 40.

### 3.5. Dynamic Changes in Leukocytes and Their Subsets’ Counts Before and After Avatrombopag Treatment

To compare the longitudinal trends of leukocyte parameters between responders and non-responders, we performed repeated-measures ANOVA with the interaction term of time × group. The results showed no significant time × group interaction for white blood cell counts (F = 0.89, *p* = 0.452), neutrophils (F = 1.02, *p* = 0.386), lymphocytes (F = 0.67, *p* = 0.573), or monocytes (F = 0.45, *p* = 0.718), as shown in Figure S2. These findings indicate that the trajectory of leukocyte subsets over the 40-day follow-up period did not differ between the two groups, further confirming that avatrombopag does not exert differential effects on white blood cell lineages regardless of platelet response status, as shown in Figure S2.

### 3.6. Safety and Surgical Outcomes

Only three patients (2.78%) received RBC transfusions (all in the non-responder group due to procedure-related bleeding, which was successfully managed). Post-recovery infections were recorded in five patients (4.63%)—all were minor wound infections or spontaneous bacterial peritonitis flares, and none were attributable to avatrombopag.

Although no thrombotic events occurred during the 40-day follow-up, this study was not powered to detect a statistically meaningful difference in thrombosis rates.

## 4. Discussion

Thrombocytopenia is a common complication in patients with CLD. Albumin solutions derived from human plasma have demonstrated clinical benefits in clinical liver disease, and surgery [20]. Afdhal et al. [4] reports that over 76% of cirrhotic patients develop thrombocytopenia. Additionally, severe thrombocytopenia (PLT counts <50 × 10^9^/L) may correlate with fatal liver disease [21,22]. The etiology of thrombocytopenia in liver disease patients is multifactorial, potentially involving hypersplenism, post-viral infection bone marrow suppression, immune mechanisms, and other factors. Close collaboration and the innovative application of immunology in the field of biosafety is becoming essential for human health.

In our study, comparative analysis of baseline characteristics between avatrombopag responders and non-responders revealed that the responder group had significantly higher baseline PLT levels than the non-responder group (*p* = 0.001). Multivariate regression confirmed baseline PLT as an independent predictive factor (*p* = 0.002), aligning with Calleja-Panero JL et al.’s findings that the baseline features of severe thrombocytopenia (PLT counts <50 × 10^9^/L) in patients with CLD (e.g., hypoalbuminemia, low PLT) correlate with treatment response [23]. Thrombocytopenia in patients with CLD primarily arises from splenic sequestration and reduced hepatic synthetic function (e.g., thrombopoietin [TPO] deficiency) [24,25]. Higher baseline PLT may reflect better-preserved megakaryocyte reserves in the bone marrow, enhancing sensitivity to avatrombopag’s thrombopoietic effects [24]. Pharmacokinetic modeling [26] further suggests that treatment response may depend on baseline hematopoietic capacity. Previous studies emphasize the critical association between avatrombopag efficacy and baseline PLT levels in CLD-related severe thrombocytopenia [25]. The responders also exhibited higher white blood cell (WBC), neutrophil (NE), and hemoglobin (HGB) levels (*p* < 0.05), potentially indicating better bone marrow function and enhanced responsiveness to thrombopoietin receptor agonists (TPO-RA) [27]. Elevated WBC and HGB levels may reflect milder bone marrow suppression or inflammatory states in patients with CLD, facilitating treatment responses [24].

In our study, correlation analysis between PLT and coagulation parameters revealed a positive association with prothrombin activity (PTA) (*p* = 0.023) and a negative correlation with the international normalized ratio (INR) (*p* = 0.020), suggesting interplay between platelet counts and coagulation status. Prior studies note that PTA and INR reflect hepatic synthetic function, with CLD-associated thrombocytopenia linked to disease severity [3,24]. However, the lack of differences in liver function markers (ALT, AST, TBIL, ALB) between groups implies avatrombopag’s efficacy depends more directly on thrombopoietic potential than hepatic function itself [11]. Gallo P et al. observed that coagulopathy in CLD often coexists with thrombocytopenia, and PLT elevation may improve coagulation [27]. Lim et al. [28] highlighted that a PLT count <50 × 10^9^/L increases the bleeding risk during invasive procedures, necessitating platelet transfusions or TPO-RA intervention. Positive correlations between PLT and HGB/WBC levels may indicate preserved hematopoietic function or milder hypersplenism-related pancytopenia. Gallo et al. [27] emphasized hypersplenism’s pivotal role in CLD-associated thrombocytopenia.

Our multivariate regression results identified baseline PLT as the sole independent predictor (*p* = 0.002), consistent with Calleja-Panero JL et al.’s real-world data showing poorer TPO-RA responses in patients with baseline PLT counts <30 × 10^9^/L [23]. Studies confirm that a higher baseline PLT increases the likelihood of achieving target PLT counts (≥50 × 10^9^/L) [25]. While WBC and HGB levels showed significance in univariate analysis, they failed in multivariate testing, possibly due to the limited sample size or confounding factors (e.g., inflammation, nutritional status). Kato et al. [29] and Shi et al. [30] noted baseline albumin ≤ 3.5 g/dL as an independent predictor of chemotherapy-induced thrombocytopenia, suggesting nutritional status may indirectly influence treatment response.

Dynamic PLT analysis post-avatrombopag treatment revealed a characteristic time window: PLT levels increased significantly by day 5, peaked at day 10, and returned to baseline by day 40. This aligns with pharmacokinetic profiles of avatrombopag, an oral TPO-RA requiring 3–5 days for onset, peaking at 7–14 days, and subsiding within 2–4 weeks post-discontinuation [3,11,13]. Huang et al. [1] reported similar trends, with repeated dosing sustaining efficacy. Tran et al. [26] highlighted its utility for short-term preoperative use to mitigate long-term risks. The non-responders exhibited minimal PLT elevation by day 5 (*p* = 0.033), failing to reach 50 × 10^9^/L, potentially due to extremely low baseline PLT counts (<20 × 10^9^/L) or severe marrow suppression [26]. Takeuchi et al. [31] proposed that TPO-RA resistance may stem from bone marrow fibrosis, reduced TPO receptor expression, or metabolic variations. Ethnic differences in efficacy among East Asian populations have also been suggested [6]. Manabe et al. [16] demonstrated that repeated lusutrombopag (a similar agent) treatment improves PLT levels, though baseline PLT counts <20 × 10^9^/L may require combination therapies. No thrombotic or hemorrhagic events occurred, consistent with long-term safety data [13,14]. Satapathy et al. [3] reported no thromboembolic complications. Avatrombopag’s selective TPO receptor activation without direct platelet stimulation likely underlies its low thrombotic risk [6]. Tran et al. [13] emphasized its safety advantages over platelet transfusions in preoperative settings.

The limitations of our study include a small sample size (18 non-responders), potentially underpowering multivariate analysis, and a lack of TPO levels/spleen volume assessments. This single-arm design without a placebo or active comparator (e.g., platelet transfusion or rhTPO) prevents a direct comparative efficacy assessment. Our response rate (83.3%) is similar to the historical controlled trial results (ADAPT-1/2: 84–88%), but a randomized controlled trial would be needed to definitively establish superiority over standard care. Future work will expand cohorts, explore combination therapies for non-responders, and evaluate long-term safety.

Given the absence of a control group and the relatively small sample, our safety findings should be interpreted with caution. The reported incidence of thromboembolic events with avatrombopag in the literature is low, but larger comparative studies are needed to confirm its thrombotic safety profile in the population with CLD.

## 5. Conclusions

This study confirms high response rates (83.33%) to avatrombopag in patients with CLD and severe thrombocytopenia. Baseline PLT emerges as a core predictor: patients with PLT counts ≥30 × 10^9^/L are more likely to benefit, potentially requiring adjunctive bone marrow reserve evaluation (e.g., megakaryocyte count) [23,24,25]. Treatment timing should align with pharmacokinetics: PLT rises by day 5, peaks around day 10, and suits short-term preoperative use [3,13]. Non-response mechanisms may involve extreme baseline thrombocytopenia or concurrent marrow suppression (e.g., hypersplenism, nutritional deficits), necessitating combination therapies (e.g., transfusions, splenic embolization) [14,27]. Avatrombopag appears to have a favorable short-term safety profile in this cohort, but the low event rate warrants larger, controlled trials to properly assess thrombotic risk. This prospective study provides the comprehensive real-world efficacy and dynamic PLT profile of avatrombopag in a large Chinese cohort with CLD undergoing various elective procedures, and it identifies baseline PLT as a practical pre-treatment predictor to guide patient selection. Avatrombopag demonstrates favorable safety without increased thrombosis risk [3,14], aligning with its pharmacodynamic profile for elective surgery preparation. These findings, consistent with the existing evidence, support individualized avatrombopag use in CLD-associated thrombocytopenia. Future studies should integrate bone marrow function assessments and optimize dosing strategies.

## Figures and Tables

**Table 2:** Logistic regression analysis of independent factors of avatrombopag treatment response in patients with chronic liver disease and metabolic abnormalities with platelet counts <50 × 10^9^/L.

Item	Univariate Logistic Regression			Multivariate Logistic Regression		
	OR	95% IC	*p* Value	OR	95% IC	*p* Value
Sex Female (%)	1.938	0.688–5.462	0.210	-	-	-
Age, mean ± SD (years)	1.023	0.973–1.075	0.374	-	-	-
WBC, median [Q1, Q3] (×10^9^/L)	0.493	0.268–0.908	** 0.023	0.584	0.310–1.102	0.097
NE median [Q1, Q3], (×10^9^/L)	0.499	0.222–1.123	0.093	-	-	-
HGB mean ± SD, (g/L)	0.976	0.957–0.995	* 0.014	0.985	0.962–1.008	0.188
PLT, median [Q1, Q3], (×10^9^/L)	0.911	0.867–0.957	*** 0.000	0.922	0.875–0.970	** 0.002
ALT, median [Q1, Q3] (U/L)	0.989	0.950–1.030	0.602	-	-	-
AST, median [Q1, Q3] (U/L)	0.974	0.931–1.019	0.246	-	-	-
TBIL, median [Q1, Q3] (umol/L)	0.999	0.974–1.025	0.940	-	-	-
DBIL, median [Q1, Q3] (umol/L)	0.994	0.944–1.047	0.824	-	-	-
ALB, median [Q1, Q3] (g/L)	0.938	0.837–1.050	0.267	-	-	-
GGT, median [Q1, Q3] (U/L)	0.997	0.987–1.008	0.613	-	-	-
Triglycerides, median [Q1, Q3] (mmol/L)	0.553	0.157–1.949	0.357	-	-	-
HDL, mean ± SD (mmol/L)	1.961	0.428–1.987	0.386	-	-	-
LDL, median [Q1, Q3] (mmol/L)	0.797	0.354–1.794	0.584	-	-	-
Blood urea nitrogen, median [Q1, Q3] (mmol/L)	0.987	0.814–1.196	0.892	-	-	-
Serum creatinine, median [Q1, Q3] (umol/L)	0.996	0.969–1.023	0.761	-	-	-
Serum uric acid, median [Q1, Q3] (umol/L)	1.000	0.997–1.004	0.817	-	-	-
Blood glucose, mean ± SD (mmol/L)	0.858	0.646–1.139	0.290	-	-	-
PTA, mean ± SD (%)	0.996	0.965–1.028	0.805	-	-	-
INR, median [Q1, Q3]	1.422	0.174–11.647	0.743	-	-	-

Note: WBCs, white blood cells; NEs, neutrophils; HGB, hemoglobin; PLT, platelet; ALT, alanine aminotransferase; AST, aspartate aminotransferase; TBIL, total bilirubin; DBIL, direct bilirubin; GGT, glutamyl transferase; HDL, high-density lipoprotein; LDL, low-density lipoprotein; PTA, prothrombin activity; INR, international normalized ratio. * *p* < 0.05, ** *p* < 0.01, *** *p* < 0.001.

## Data Availability

After publication, the non-private data of patients can be available from the corresponding author on reasonable request.

## Supplementary Materials

The following supporting information can be downloaded at https://www.mdpi.com/article/10.3390/jcm15145715/s1.

## Abbreviations

The following abbreviations are used in this manuscript:

CLD	chronic liver disease
TPO	thrombopoietin
WBC	white blood cells
NE	neutrophils
HGB	hemoglobin
PLT	platelet
ALT	alanine aminotransferase
AST	aspartate aminotransferase
TBIL	total bilirubin
DBIL	direct bilirubin
GGT	glutamyl transferase
HDL	high-density lipoprotein
LDL	low-density lipoprotein
PTA	prothrombin Activity
INR	international normalized ratio
