# Supplementary material for: Efficacy and Safety of Avatrombopag in Patients with Chronic Liver Disease and Thrombocytopenia Undergoing Elective Surgery
Source: J Clin Med. 2026 Jul 21;15(14):5715. doi: 10.3390/jcm15145715 (PMC13412254; doi:10.3390/jcm15145715)
Supplement: Supplementary file 1 [file jcm-15-05715-s001.zip › jcm-4390692-supplementary.pdf]

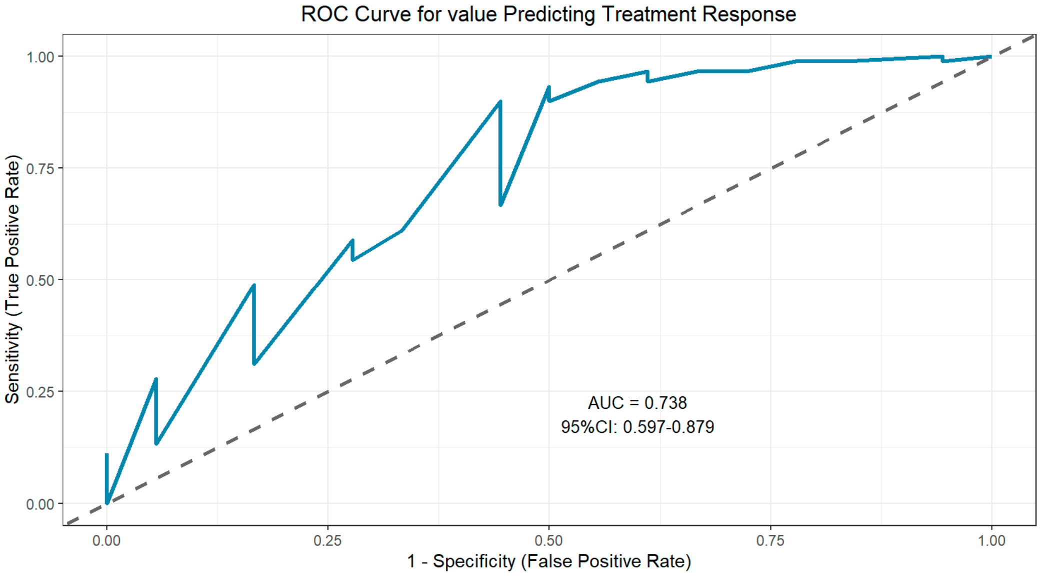

Figure S1. The ROC curve analysis of baseline platelet count as an independent factor.

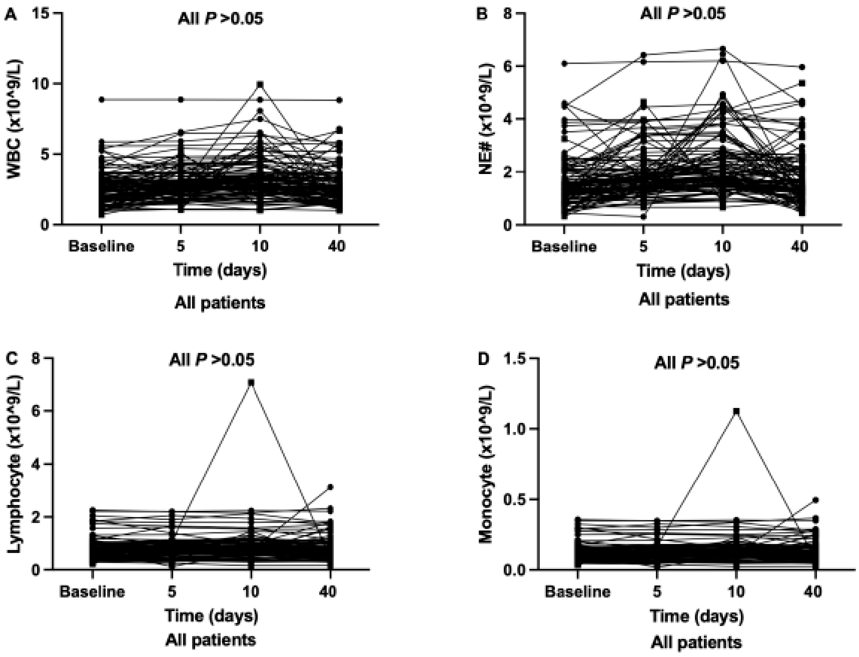

Figure S2. Peripheral white blood cell (WBC) (A), neutrophil (NE#) (B), lymphocyte (C), and monocyte (D) counts were measured at baseline, on treatment day 5, on procedure day (approximately day 10), and at follow-up day 40 in all patients.
